# Fc-null anti-PD-1 monoclonal antibodies deliver optimal checkpoint blockade in diverse immune environments

**DOI:** 10.1136/jitc-2021-003735

**Published:** 2022-01-11

**Authors:** Julia Moreno-Vicente, Jane E Willoughby, Martin C Taylor, Steven G Booth, Vikki L English, Emily L Williams, Christine A Penfold, C Ian Mockridge, Tatyana Inzhelevskaya, Jinny Kim, H T Claude Chan, Mark S Cragg, Juliet C Gray, Stephen A Beers

**Affiliations:** 1 Antibody and Vaccine Group, Centre for Cancer Immunology, Cancer Sciences, University of Southampton, Southampton, UK; 2 Cancer Research UK Cambridge Institute, University of Cambridge, Cambridge, UK

**Keywords:** immunotherapy, programmed cell death 1 receptor, antibodies, neoplasm

## Abstract

**Background:**

Despite extensive clinical use, the mechanisms that lead to therapeutic resistance to anti-programmed cell-death (PD)-1 monoclonal antibodies (mAbs) remain elusive. Here, we sought to determine how interactions between the Fc region of anti-PD-1 mAbs and Fcγ receptors (FcγRs) affect therapeutic activity and how these are impacted by the immune environment.

**Methods:**

Mouse and human anti-PD-1 mAbs with different Fc binding profiles were generated and characterized in vitro. The ability of these mAbs to elicit T-cell responses in vivo was first assessed in a vaccination setting using the model antigen ovalbumin. The antitumor activity of anti-PD-1 mAbs was investigated in the context of immune ‘hot’ MC38 versus ‘cold’ neuroblastoma tumor models, and flow cytometry performed to assess immune infiltration.

**Results:**

Engagement of activating FcγRs by anti-PD-1 mAbs led to depletion of activated CD8 T cells in vitro and in vivo, abrogating therapeutic activity. Importantly, the extent of this Fc-mediated modulation was determined by the surrounding immune environment. Low FcγR-engaging mouse anti-PD-1 isotypes, which are frequently used as surrogates for human mAbs, were unable to expand ovalbumin-reactive CD8 T cells, in contrast to Fc-null mAbs. These results were recapitulated in mice expressing human FcγRs, in which clinically relevant hIgG4 anti-PD-1 led to reduced endogenous expansion of CD8 T cells compared with its engineered Fc-null counterpart. In the context of an immunologically ‘hot’ tumor however, both low-engaging and Fc-null mAbs induced long-term antitumor immunity in MC38-bearing mice. Finally, a similar anti-PD-1 isotype hierarchy was demonstrated in the less responsive ‘cold’ 9464D neuroblastoma model, where the most effective mAbs were able to delay tumor growth but could not induce long-term protection.

**Conclusions:**

Our data collectively support a critical role for Fc:FcγR interactions in inhibiting immune responses to both mouse and human anti-PD-1 mAbs, and highlight the context-dependent effect that anti-PD-1 mAb isotypes can have on T-cell responses. We propose that engineering of Fc-null anti-PD-1 mAbs would prevent FcγR-mediated resistance in vivo and allow maximal T-cell stimulation independent of the immunological environment.

## Introduction

Programmed cell-death (PD)-1 is an inhibitory coreceptor largely expressed on activated CD8 T cells, which has been shown to play a critical role in downregulating tumor-specific T-cell responses in cancer.^1^ The success achieved in some advanced adult malignancies^2 3^ with monoclonal antibodies (mAbs) that block PD-1 ligation has led to this strategy becoming a central pillar in the treatment of cancer, with currently four anti-PD-1 mAbs approved in the clinic. Nevertheless, the majority of patients do not respond to anti-PD-1, and hence focus has turned to elucidating the mechanisms that drive primary resistance.

Choice of isotype is critical for therapeutic mAbs, as IgG isotypes have distinct abilities to engage effector mechanisms.^4^ This largely reflects their differential binding to Fc gamma receptors (FcγRs), a class of transmembrane glycoproteins involved in regulating immune activation.^5^ FcγRs are composed of a set of activating receptors (in mice, FcγRI, FcγRIII and FcγRIV; in humans (h) hFcγRI, hFcγRIIa, hFcγRIIc, hFcγRIIIa and hFcγRIIIb) and a sole inhibitory receptor (FcγRII or hFcγRIIb), with the balance between activating and inhibitory receptor engagement setting a threshold for cellular activation.^6^ Although initially conceived that mAbs used for cancer therapy required engagement of FcγRs expressed on effector cells, it has become clear that FcγR engagement requirement varies according to mAb class. While tumor-targeting mAbs (eg, anti-CD20) require activating FcγR engagement to trigger effector mechanisms,^7–9^ inhibitory FcγRIIb binding has been demonstrated to optimally deliver agonistic activity for a range of costimulatory mAbs.^10–13^

In marked contrast, anti-PD-1 mAbs are understood to act predominantly via receptor blockade, and hence expected to not require FcγR engagement. In keeping with this, the four clinically approved anti-PD-1 mAbs were designed as hIgG4 to minimize FcγR binding.^14^ However, antigen-bound hIgG4 mAbs are reported to bind to both activating and inhibitory FcγRs,^15 16^ implying that anti-PD-1 mAbs could trigger effector mechanisms, potentially impacting efficacy. Although previous studies support that FcγR engagement can modulate the antitumor activity of anti-PD-1 mAbs,^17 18^ the extent to which T-cell responses are modulated in different immune settings is not understood.

Here, we examined how the Fc requirements for anti-PD-1 mAbs were impacted by the immune environment; first, in an immunization setting, using the model antigen ovalbumin (OVA), and then in the context of immunologically hot vs cold tumors. To this end, we compared the immunogenic MC38 model, which bears a high tumor mutational burden (TMB),^19^ with the 9464D pediatric neuroblastoma model.^20 21^ Pediatric cancers represent a paradigm of immunologically cold tumors with a low mutational load, limited T-cell infiltration, and generally poor responsiveness to anti-PD-1/PD-L1.^22^ However, like many adult cancers, there is evidence of PD-1/PD-L1 expression in pediatric tumors,^23 24^ supporting the use of preclinical models to better understand how to target PD-1.

We found that the impact of FcγR binding was different in immunization vs tumor settings. Notably, anti-PD-1 with high (mIgG2a) or reduced (mIgG1) affinity for FcγRs were unable to expand endogenous or adoptively transferred OVA-reactive CD8 T cells. In contrast, Fc-null (mIgG1-N297A) mAb synergized with anti-CD40 to enhance CD8 T-cell expansion and effector phenotype. Results obtained with murine mAbs were translated into a humanized system,^25^ with Fc-null anti-PD-1 (hIgG4-FALA)^26^ outperforming wild-type hIgG4 to enhance endogenous CD8 T-cell expansion in mice bearing hFcγRs. In the context of cancer, both mIgG1 and mIgG1-N297A significantly improved survival and immune activation in MC38 tumors, whereas mIgG2a led to depletion of activated CD8 tumor-infiltrating lymphocytes (TILs) and completely abrogated therapeutic activity. We conclude that Fc:FcγR interactions represent a mechanism of resistance to PD-1 blockade that varies with immune environment, and propose that Fc-null mAbs have greater potential for clinical benefit.

## Materials and methods

### Animals and cells

Animals were bred and maintained by the Biomedical Research Facility (University of Southampton) in accordance with Home Office guidelines. C57BL/6 and OVA-TCR-I (OT-I)^27^ mice were purchased from Charles River. Mice bearing all human FcγR^25^ were provided by Regeneron Pharmaceuticals. Females between 8 and 14 weeks of age were used throughout. Sample size was guided by previous experience and preliminary data. Treatment cohorts were randomized to ensure tumors were size-matched at the start of each experiment. Investigators performing tumor measurements were not blinded. For OT-I transfer experiments, mice that showed less than 2% of SIINFEKL-specific CD8 T cells at day 3 after mAb treatment were considered outliers and were excluded from analysis.

MC38 cells^19^ were maintained in complete RPMI1640 supplemented with 1% glutamine/pyruvate, penicillin-streptomycin, 10% FCS. Mouse neuroblastoma 9464D cells were cultured as previously described.^20^

### Antibodies

Parental anti-mouse PD-1 (EW1-9) rIgG1 was raised using conventional hybridoma technology.^28^ Mouse and human anti-PD-1 EW1-9 mIgG1, mIgG2a, mIgG1-N297A, hIgG4 and hIgG4-FALA^26^ were constructed as previously described.^12 29^ Antibodies generated in-house were produced from hybridoma or CHO-K1 cells and purified on Protein A with purity assessed by electrophoresis (Beckman EP system) and lack of aggregation by size exclusion chromatography and high-performance liquid chromatography (SEC-HPLC). All preparations were endotoxin low (<1 ng/mg) as determined using Endosafe-PTS system.

### Surface plasmon resonance

Protein interactions were assayed using Biacore T100 (GE Healthcare Life Sciences). Briefly, His-tagged recombinant proteins (R&D Systems) were immobilized at 5000 response units (RU) to the flow cells of CM5 sensor chips using a His Capture Kit (GE Healthcare Life Sciences). To assess binding to PD-1 or FcγRs, anti-PD-1 mAbs were injected through the flow cells at 0.16–100 nM or 6.2–500 nM, respectively, in HBS-EP+ running buffer (hepes-buffered saline, 0.3 mM EDTA, 0.05% surfactant P20) and flow rate 30 µL/min. For cross-blocking experiments, mAbs were injected at 100 µL/min to saturate the chip. Fc-recombinant PD-L1 or PD-L2 (25 µg/mL; R&D Systems) were then injected at 25 µL/min. Background binding to control flow cells was subtracted from measurements. Affinity constants were derived by analysis of association and dissociation using a bivalent binding model (Bioevaluation software; Biacore).

### Binding assays

HEK293F cells transiently transfected with mPD-1 were incubated with indicated concentrations of anti-PD-1 mAbs at 4°C for 30 min prior to staining with PE-labeled anti-mouse Fc or APC-labeled anti-rat Fc secondary antibodies (Jackson Laboratories). For competition assays, mPD-1-expressing HEK293F were incubated with 1 µg/mL AF488-labeled rat anti-PD-1 mAb and graded concentrations of mouse anti-PD-1 isotypes at 4°C for 40 min. Samples were acquired on FACSCalibur and data analyzed with FCS Express.

### Antibody half-life

C57BL/6 mice were administered 250 µg of mouse anti-PD-1 intraperitoneally (i.p) and blood collected at indicated time points. To determine antibody concentration, transfected mPD-1-expressing HEK293F were incubated with a 1/10,000 serum dilution or known dilution range of anti-PD-1 for 30 min at 4°C. Samples were stained with PE-labeled anti-mouse Fc secondary antibody at 4°C for 30 min and acquired on FACSCalibur and data analyzed with FCS Express.

### T cell suppression assay

Purified CD8 T cells were plated in the presence of 1 µg/mL plate-bound anti-CD3 (clone 145–2 C11; in-house) plus 5 µg/mL mPD-L1-Fc or irrelevant Fc-recombinant proteins (R&D Systems). Mouse anti-PD-1 or irrelevant mAbs were added (5 µg/mL) and cultures incubated for 4 days at 37 °C. For the last 16 hours of culture, 1 µCi/well [^3^H]-thymidine (Perkin Elmer) was added and a scintillation β-counter used to measure DNA radioactivity.

### Antibody-dependent cellular phagocytosis

Phagocytosis assay was performed as described previously.^30 31^ Briefly, purified CD3 T cells were activated with 1 µg/mL plate-bound anti-CD3 and used as targets. Bone marrow-derived macrophages (BMDMs) were generated,^30 31^ polarized with 2 ng/mL IFN-γ (Peprotech) and 50 ng/mL lipopolysaccharide (LPS; Sigma-Aldrich) overnight and used as effector cells. The percentage of carboxyfluorescein succinimidyl ester+ (CFSE+), F4/80-AF647+ double-positive cells out of F4/80-AF647+ macrophages was assessed by flow cytometry and presented as percentage of phagocytosis.

### OVA-specific immune responses

Splenocytes from transgenic OT-I mice were harvested and 10^5^ naïve OT-I T cells transferred intravenously to age and sex matched C57BL/6 recipients. One day later, mice were challenged i.p with 5 mg OVA (Sigma) in combination with the indicated amount of anti-CD40 (clone 3/23; in-house) plus 200 µg anti-PD-1, as specified in figure legends. Following contraction of primary responses, re-challenge was performed by intravenous injection of 30 nm SIINFEKL peptide (Sigma-Aldrich). Blood samples were collected at specified timepoints and stained with mCD8-PerCP-Cyanine5.5 (53–6.7), PE-labeled H2K^b^-SIINFEKL tetramer (in-house), CD62L-Pacific Blue (MEL-14) or isotype control (RTK2758; both BioLegend), CD44-FITC (IM7), mPD-1-PerCP-eFluor710 (RMP1-30) or isotype controls (eB149/10H5) (all eBioscience). Samples were acquired on a FACSCanto II and analyzed with FlowJo Software.

### Immunotherapy

C57BL/6 mice were challenged with 5×10^5^ MC38 or 9464D cells subcutaneously on day 0. When tumors reached 50 mm^2^, mice received 200 µg anti-PD-1 or isotype-matched irrelevant controls i.p, followed by two further administrations 4 and 7 days later. Tumor sizes were monitored three times/week using calipers and mice culled at humane endpoint (225 mm^2^) or if animal welfare was compromised.

### Immune phenotyping

One day after final mAb administration, mice were euthanized and spleens and tumors harvested. Tumors were enzymatically digested (Liberase TL and DNase; Roche) for 20 min at 37 °C. Spleens and tumors were mechanically disaggregated and passed through a cell strainer to obtain single-cell suspensions. Prior to staining, cells were incubated with 10 µg/mL of 2.4G2 mAb (in-house) for 15 min on ice (except where FcγR staining was performed). Cell suspensions were stained with appropriate antibodies for 30 min on ice and fixed with Erythrolyse Red Blood Cell Lysing buffer (BioRad). For intracellular staining, the anti-Mouse/Rat Foxp3 Staining Set (BD Biosciences) was used according to manufacturer’s instructions.

Antibodies were purchased from eBioscience unless otherwise stated: mCD45.2-PE-Cyanine7 (104 RUO), mCD4-eFluor450 (GK1.5), mCD8-PerCP-Cyanine5.5 (53–6.7), FoxP3-PE (FJK-16s), CD11c-eFluor450 (N418), Ly-6C-PerCP-Cyanine5.5 (HK1.4), Ly-6G-PE-Cyanine7 (RB6-8C5), CD11b-PE (M1/70), MHC-II I-A/I-E-V500 (M5/114.15.2), F4/80-APC (Cl:A3-1; in-house), mPD-L1-PE (MIH5) or isotype control (eBR2a), mPD-1-APC (RMP1-30) or isotype control (eB149/10H5), CD44-FITC (IM7) or isotype control (eB149/10H5), CD62L-APC-Cyanine7 (MEL-14; BioLegend) or isotype control (RTK2758; BioLegend). Additionally, staining of mouse FcγRs was performed with FITC-labeled mAbs (in-house) as previously described.^32^ Samples were acquired on a FACSCanto II and analyzed with FlowJo Software.

### Statistical analysis

Statistical analysis was performed using GraphPad Prism Software. Graphs show mean±SD unless otherwise stated. Statistical differences between groups were assessed by unpaired two-tailed Student’s t test or one-way analysis of variance (Tukey’s multiple comparison test). Kaplan-Meier curves were produced from survival experiments and analyzed by log rank (Mantel-Cox) test. Differences were considered statistically significant when p<0.05. Significance denoted as follows: *p<0.05, **p<0.01 and ***p<0.001.

## Results

### Engineered mouse anti-PD-1 mAbs retain binding and in vitro function

**Figure 1:**
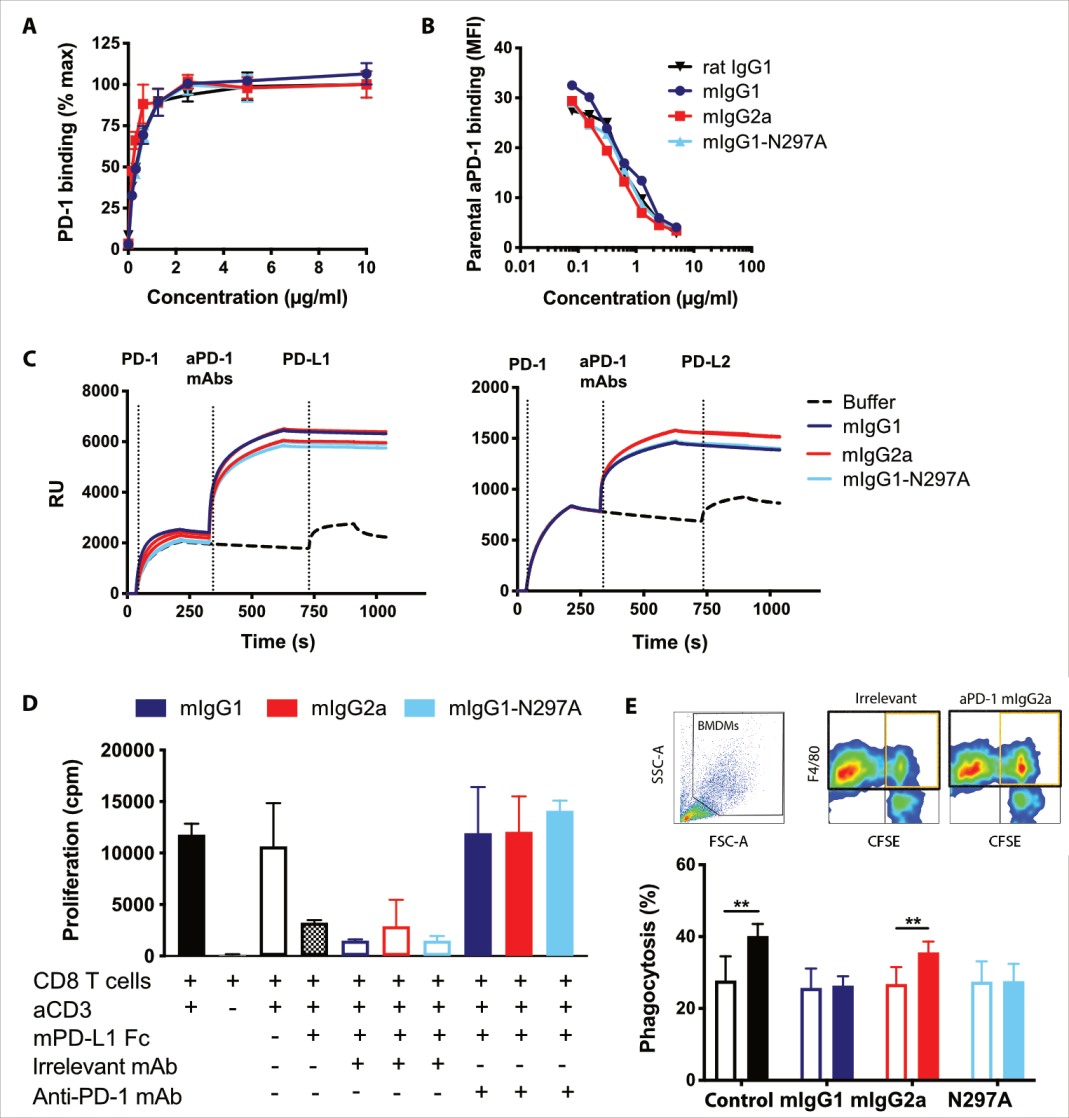
Engineered mouse anti-PD-1 mAbs retain equivalent in vitro binding properties and function. (A) Mouse PD-1-transfected HEK293F cells were incubated with the indicated anti-PD-1 mAb isotypes at a range of concentrations prior to staining with a PE- or APC-labeled secondary antibodies. Data show mean fluorescence intensity (MFI) as a percentage of maximum. (B) Cells were incubated with anti-PD-1 mAb as in (A) in the presence of 1 µg/mL AF488-conjugated rat anti-PD-1 mAb. Data are presented as MFI of the rat anti-PD-1 relative to the concentration of competitive mouse mAb. Data in (A) and (B) show one representative experiment of two. Bars represent mean±SEM of triplicates. (C) Surface plasmon resonance analysis illustrating anti-PD-1 mAb binding to mouse PD-1 and blockade of PD-L1/2. Mouse anti-PD-1 mAbs (100 µg/mL) were passed over His-tagged PD-1 captured with an anti-His mAb. Recombinant PD-L1 or PD-L2-Fc were passed over (25 µg/mL) to demonstrate PD-1 binding or blockade. (D) Purified CD8 T cells from C57BL/6 mice were incubated with 1 µg/mL plate-bound anti-CD3, 5 µg/mL plate-bound PD-L1-Fc or irrelevant controls, and 5 µg/mL soluble mouse anti-PD-1 mAbs or irrelevant isotypes. Proliferation was assessed by [3H]-thymidine incorporation. Data show combined means from two independent experiments. Bars represent mean±SD. (E) Activated CFSE-labeled murine splenic T cells were opsonized with anti-PD-1 isotypes (filled bars) or irrelevant controls (open bars) prior to coculture with BMDMs. Experiment performed twice in triplicates. Bars show mean±SD. Student T-test, **p<0.01. BMDM, bone marrow-derived macrophages; CFSE, carboxyfluorescein succinimidyl ester; mAb, monoclonal antibodies; PD-1, programmed cell-death.

To elucidate the importance of the anti-PD-1 Fc-region, three mouse isotypes were produced by engineering the parental rat IgG1 constant region to mIgG1, mIgG2a or the Fc-null variant mIgG1-N297A. Surface plasmon resonance (SPR) confirmed the expected FcγR binding pattern, with mIgG1 and mIgG2a displaying low and high activating to inhibitory FcγR-binding ratio (A:I), respectively,^6^ while abrogation of N-glycosylation by N297A mutation prevented appreciable binding to all FcγRs (online supplemental figure S1A,^33^). All three isotypes demonstrated equivalent binding to PD-1 (figure 1A), with comparable ability to compete for cell-surface PD-1 (figure 1B) and similar avidity (online supplemental figure S1B). Further SPR analysis confirmed that all mouse isotypes blocked PD-1 interaction with its ligands, PD-L1 and PD-L2 (figure 1C). In agreement with this, PD-L1 mediated inhibition of CD8 T-cell proliferation was rescued by all mouse isotypes (figure 1D). These experiments confirmed that modification of the Fc-region did not alter binding to PD-1 or the ability to block ligand-induced T-cell suppression.

10.1136/jitc-2021-003735.supp1 Supplementary data

Antibody half-life is a principal determinant of in vivo mAb activity. We therefore established that our anti-PD-1 isotypes retained similar half-lives (online supplemental figure S1C), confirming that therapeutic efficacy would not be affected by differences in bioavailability. To understand the potential impact of FcγR engagement by anti-PD-1 mAbs, we first examined the capacity of each isotype to trigger effector functions in vitro. Phagocytosis of mAb-opsonized target cells by FcγR-expressing myeloid cells is a well-established mechanism of action for tumor-targeting mAbs.^8^ Importantly, mAbs targeting T-cell receptors such as CD25^34^ or 4-1BB^28^ have also been shown to cause depletion of T-cell populations expressing high levels of target receptor. Given the reportedly high expression of PD-1 by TILs, we sought to establish whether engagement of FcγRs by anti-PD-1 could also cause phagocytosis. For this, activated primary T cells expressing PD-1 (online supplemental figure S1D) were cocultured with BMDMs in the presence of mouse anti-PD-1 mAbs. This demonstrated that opsonization with the high A:I ratio mIgG2a, but not low mIgG1 or Fc-null mIgG1-N297A isotypes, resulted in increased phagocytosis of PD-1-expressing CD8 T cells by BMDMs (figure 1E). This indicates that engagement of activating FcγRs by anti-PD-1 could mediate the phagocytosis of activated TILs in vivo, reducing therapeutic activity.

### Fc-null anti-PD-1 mAbs enhance expansion and effector phenotype of OT-I cells

Next, we sought to investigate the ability of anti-PD-1 mAbs to expand antigen-specific T-cell responses in vivo. Transfer experiments using OT-I cells allow the monitoring of antigen-specific CD8 expansion and phenotype following immunization with their cognate antigen OVA.^27^ To provide the second signal required for T-cell activation, a costimulatory mAb targeting CD40 was used alongside OVA. The combination of anti-CD40 with OVA was able to induce a dose dependent CD8 expansion, accompanied by concomitant upregulation of PD-1 (online supplemental figure S2). Because of this, we hypothesized that PD-1 blockade could further increase T-cell expansion by releasing PD-1-mediated inhibition. Therefore, a dose of anti-CD40 that resulted in suboptimal OVA-specific CD8 expansion (10 µg, CD40^Lo^) was selected for subsequent combination experiments.

**Figure 2:**
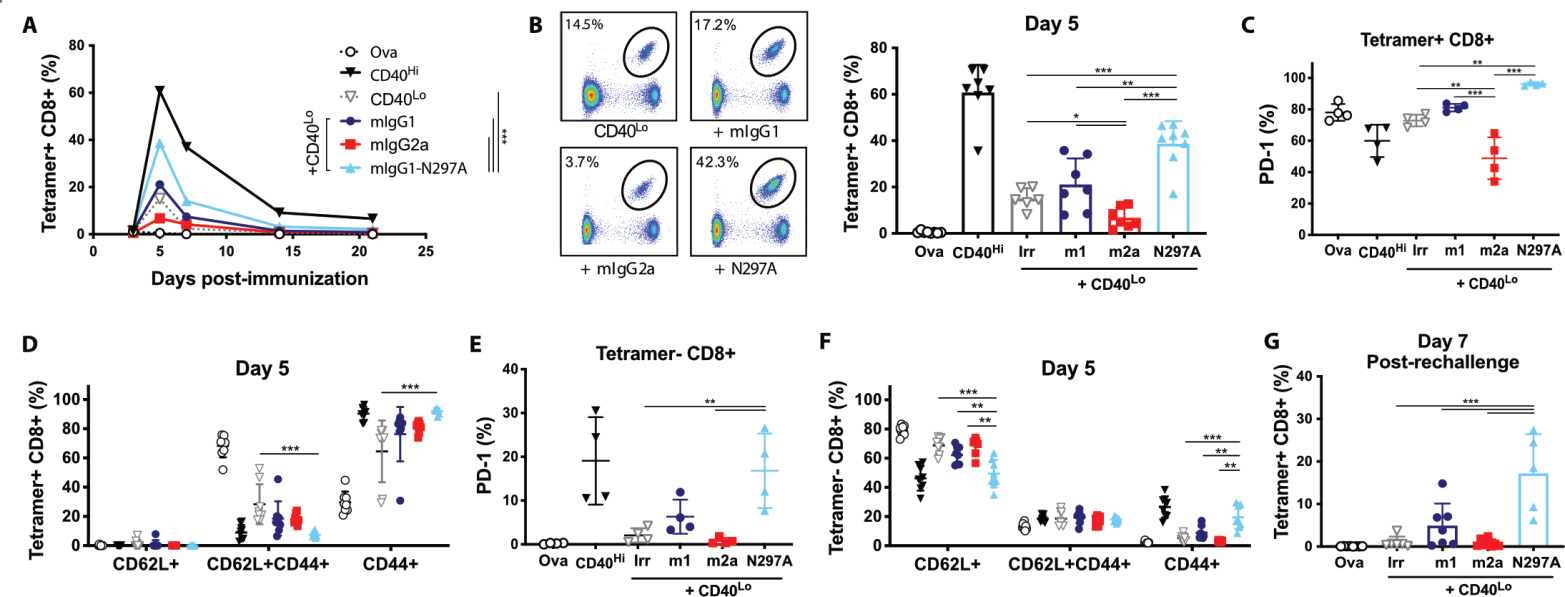
Expansion of OT-I cells is enhanced by mIgG1-N297A but impaired with mIgG2a anti-PD-1 mAbs. (A–F) Groups of C57BL/6 mice received OT-I cell transfer prior to intraperitoneal injection with 5 mg OVA alone or plus the indicated treatments on day 0. (A) Kinetics of SIINFEKL-specific CD8 T-cell expansion (shown as % of lymphocytes) after treatment with 100 µg anti-CD40 (CD40^Hi^), 10 µg anti-CD40 plus irrelevant mAbs (CD40^Lo^) or CD40^Lo^ plus anti-PD-1 isotypes. (B) Example plots and percentage of SIINFEKL-specific CD8 T cells at day 5. (C–D) Expression of PD-1 (C) and frequency of CD62L+, CD44+ and double positive cells (D) in SIINFEKL-specific CD8 T cells at day 5. (E, F) Expression of PD-1 (E) and frequency of CD62L+, CD44+ and double positive cells (F) in tetramer negative CD8 T cells at day 5. (G) Percentage of SIINFEKL-specific CD8 T cells (as % of lymphocytes) at day seven following rechallenge with SIINFEKL peptide. Experiment performed twice, n=6–8 mice per group. Data in (C, E) show one representative experiment of two. Bars represent mean±SD. *P<0.05, **p<0.01, ***p<0.001 (one-way ANOVA). ANOVA, analysis of variance; mAbs, monoclonal antibodies; PD-1, programmed cell-death; OVA, ovalbumin.

In combination with CD40^Lo^, anti-PD-1 mIgG1-N297A significantly increased OT-I expansion throughout the primary response (AUC; figure 2A) compared with other treatment groups. Similarly, this isotype markedly increased the percentage of SIINFEKL-specific CD8s at the peak of the response (figure 2B). Importantly, this population of expanded CD8s were CD44+CD62L−, highlighting their activation and acquisition of an effector-like phenotype, and displayed increased PD-1 expression (figure 2C–D). In contrast, treatment with mIgG2a decreased the percentage of SIINFEKL-specific (figure 2B) and PD-1+CD8 T cells (figure 2C) compared with controls. In line with our in vitro data, this indicated a potential depletion of PD-1-expressing effector cells by mIgG2a through engagement of activating FcγRs.

Interestingly, we found evidence of CD8 activation in the tetramer negative subset following treatment with mIgG1-N297A. An increase in the percentage of CD44+CD62L− effectors was noted, which was driven by the activation of newly primed naïve (CD62L+) T cells (figure 2F). Further to this, we observed a parallel increase in the percentage of tetramer negative CD8s expressing PD-1, possibly marking the acquisition of an activated phenotype (figure 2E). These findings demonstrate that lack of FcγR engagement by anti-PD-1 mAbs enhances their ability to expand primary responses and may also serve to broaden the response to non-dominant epitopes.

We next interrogated the capacity of OT-I cells primed in the presence of anti-PD-1 to generate memory responses. In mIgG1-N297A treated mice, rechallenge with SIINFEKL peptide led to strong recall responses similar to those of the primary challenge (figure 2G). Despite the lack of significant expansion during the primary response to OVA, mIgG1 was still able to enhance the generation of antigen-specific CD8s on rechallenge, although less effectively than the mIgG1-N297A isotype.

### Fc-null anti-PD-1 mAbs augment endogenous CD8 T-cell immunity to OVA

**Figure 3:**
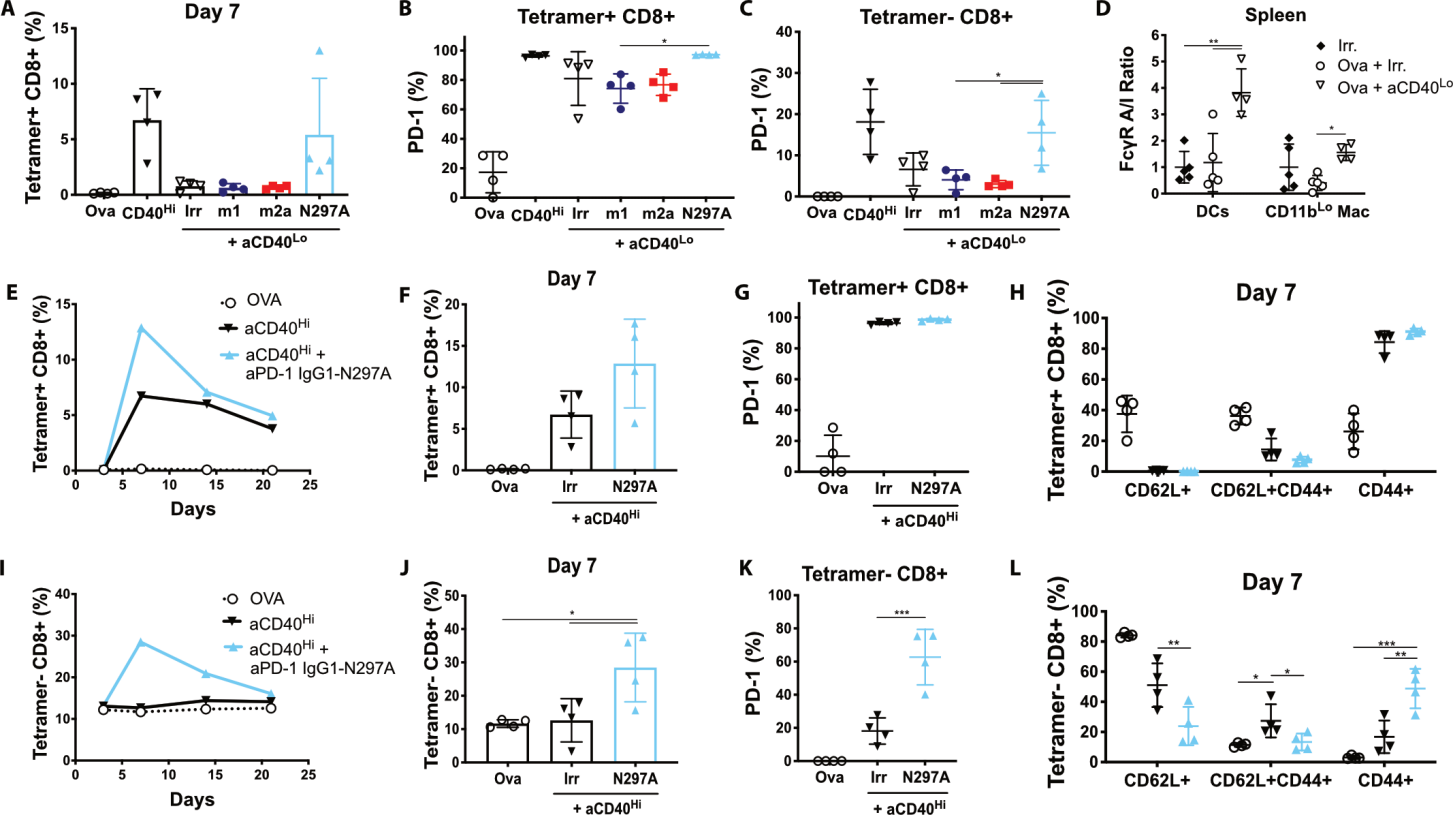
Mouse anti-PD-1 IgG1-N297A but not mIgG1 or mIgG2a enhances endogenous CD8 T-cell responses to OVA. (A–C) C57BL/6 mice received 5 mg OVA alone or in combination with 100 µg anti-CD40 (CD40^Hi^), 10 µg anti-CD40 plus irrelevant mAbs (CD40^Lo^) or CD40^Lo^ plus anti-PD-1 isotypes. (A, B) Percentage of (A) and PD-1 expression in (B) SIINFEKL-specific CD8 T cells at day 7. (C) Expression of PD-1 in tetramer negative CD8 T cells at day 7. (D) Mice received the indicated treatments and spleens were harvested 3 days later to assess expression of FcγRs in DCs and tissue-resident macrophages (CD11b^Lo^ Mac). A:I ratios were calculated by dividing the sum of MFI from FcγRI, III and IV by that of FcγRII. E-L) C57BL/6 mice received 5 mg OVA alone or in combination with 100 µg anti-CD40 plus irrelevant mAbs (CD40^Hi^) or plus anti-PD-1 mIgG1-N297A mAb. (E) Kinetics of SIINFEKL-specific CD8 T-cell expansion (shown as % of CD8 cells). (F) Percentage of SIINFEKL-specific CD8 T cells at day 7. (G–H) Expression of PD-1 (G) and frequency of CD62L+, CD44+ and double positive cells (H) in SIINFEKL-specific CD8 T cells at day 7. (I) Kinetics of tetramer negative CD8 T-cell expansion (shown as % of lymphocytes). (J) Percentage of tetramer negative CD8 T cells at day 7. (K–L) Expression of PD-1 (K) and frequency of CD62L+, CD44+ and double positive cells (L) in tetramer negative CD8 T cells at day 7. Experiment performed once, N=4 mice per group. Bars represent mean±SD, **p<0.05, **p<0.01, ***p<0.001 (One-way ANOVA). A/I Ratio, activating to inhibitory FcγR-binding ratio; ANOVA, analysis of variance; DCs, dendritic cells; FcγRs, Fcγ receptors; mAbs, monoclonal antibodies; MFI, mean fluorescence intensity; PD-1, programmed cell-death; OVA, ovalbumin.

To validate our findings in an endogenous setting, we performed the same experiment in the absence of OT-I cells. While in this setting CD40^Lo^ was not sufficient to expand SIINFEKL-specific CD8s with either anti-PD-1 mIgG1 and mIgG2a, the combination with mIgG1-N297A enhanced the expansion of SIINFEKL-specific CD8s (figure 3A) and the percentage of PD-1+ cells (figure 3B). Nevertheless, PD-1 upregulation was more pronounced in the tetramer negative CD8 T-cell subset (figure 3C), in keeping with the results observed following OT-I transfers (figure 2E). These results further indicated that engagement of FcγRs by anti-PD-1 impairs T-cell expansion and reduces the potency of blocking the inhibitory signal delivered by PD-1 ligation.

Notably, mIgG1 also abrogated the activity of PD-1 blockade in this context, despite its low A:I ratio (online supplemental figure S1A) and the fact that this isotype did not induce phagocytosis in vitro (figure 1F). However, other studies have shown that mIgG1 mAbs can still cause depletion of target T cells in the absence of FcγRII,^28^ highlighting the importance of the relative expression of activating to inhibitory FcγRs for mAb-mediated effector functions. We therefore hypothesized that concurrent delivery of OVA plus anti-CD40 could activate myeloid cells and up-regulate activating FcγRs, thereby increasing mIgG1 A:I ratio and favoring its effector functions. Following treatment with OVA plus CD40^Lo^, dendritic cells (DCs) and resident macrophages in the spleen displayed an increased A:I ratio (figure 3D). Upregulation of FcγRI and IV, together with decreased FcγRII expression, were responsible for this increase (online supplemental figure S3). Therefore, these results support the contention that the poor CD8 expansion mediated by mIgG1 could too be caused by the engagement of activating FcγRs and depletion of PD-1-expressing T cells.

Given that the percentage of SIINFEKL-specific CD8s expanded with 10 µg anti-CD40 was minimal, we sought to investigate whether the combination of 100 µg anti-CD40 (CD40^Hi^) with mIgG1-N297A would lead to more profound changes in T-cell responses. Analogous to the findings with lower doses, combination with mIgG1-N297A augmented the expansion of SIINFEKL-specific CD8s at the peak of the response (figure 3E, F). In this context, the frequency of PD-1 + and effector cells was already maximal with CD40^Hi^ alone (figure 3G, H).

PD-1 is expressed on all activated CD8 T cells, and therefore, anti-PD-1 has the potential to reinvigorate multiple T-cell specificities. While signs of activation were noted on tetramer negative OT-I cells after treatment with mIgG1-N297A (figure 2E,F), it is possible that this was due to a bystander effect from the initial large expansion of SIINFEKL-reactive OT-I cells (figure 2A) rather than direct PD-1 blockade on the tetramer negative subset. Therefore, we sought to elucidate whether CD8 T cells with other specificities could also be expanded and activated in the absence of large numbers of high-affinity OT-I cells. In the setting of an endogenous response to OVA, CD40^Hi^ was unable to expand tetramer negative CD8s; however, addition of anti-PD-1 mIgG1-N297A effectively expanded this population (figure 3I, J). Similar to OT-I transfer experiments, this expansion was accompanied by an upregulation of PD-1 and an increase in effector-like (CD44+CD62L-) tetramer negative T cells (figure 3K, L), providing evidence of the ability of anti-PD-1 mIgG1-N297A to activate multiple T-cell specificities in the absence of adoptive T-cell transfer.

### Human Fc-null anti-PD-1 mAb enhances the endogenous CD8 T-cell response to OVA in hFcγR-expressing mice

**Figure 4:**
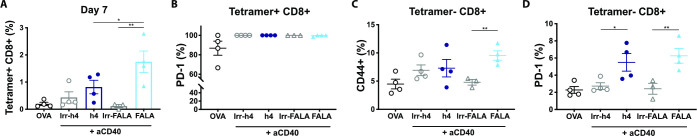
Human anti-PD-1 IgG4-FALA enhances endogenous CD8 T-cell responses to OVA in hFcγR-expressing mice. Human FcγR-expressing mice received (i.p) 5 mg OVA alone or in combination with 200 µg anti-CD40 (mIgG1), 200 µg anti-CD40 plus 250 µg irrelevant mAbs (AT171-2 hIgG4 or AT171-2 hIgG4-FALA; in-house) or 200 µg anti-CD40 plus 250 µg anti-PD-1 mAbs (EW1-9 hIgG4 or hIgG4-FALA). (A, B) Percentage of (A) and PD-1 expression in (B) SIINFEKL-specific CD8 T cells at day 7. (C, D) Expression of PD-1 (C) and frequency of CD44+ cells in tetramer negative CD8 T cells at day 7. Representative of two independent experiments, N=4 mice per group. Bars represent mean±SD, *p<0.05, **p<0.01, (One-way ANOVA). ANOVA, analysis of variance; FALA, F234A-L235A mutation; FcγR, Fcγ receptors; PD-1, programmed cell-death.

Despite being powerful tools to study Fc:FcγR interactions, mouse isotypes do not fully recapitulate the FcγR binding pattern of human IgGs.^5^ To ascertain that clinically relevant anti-PD-1 human isotypes would retain similar Fc requirements to their murine counterparts, we investigated the effect of anti-PD-1 hIgG4 and its Fc-null counterpart (hIgG4-FALA) on OVA-specific T-cell responses in the context of FcγR humanized mice.^25^ Here, hIgG4-FALA significantly enhanced CD8 T-cell expansion compared with its Fc-competent counterpart and irrelevant controls (figure 4A), despite all treatment groups displaying maximal PD-1 expression in SIINFEKL-specific CD8 T cells (figure 4B). Moreover, hIgG4-FALA promoted an effector-like phenotype in tetramer negative CD8 T cells, with increased percentage of CD44+CD62L− cells as well as PD-1+ (figure 4C, D). Therefore, the results obtained with murine anti-PD-1 isotypes were recapitulated by human mAbs, indicating that similar mechanisms could hinder PD-1 blockade in the clinic.

### Fc-regions of anti-PD-1 mAbs determine antitumor efficacy in MC38

**Figure 5:**
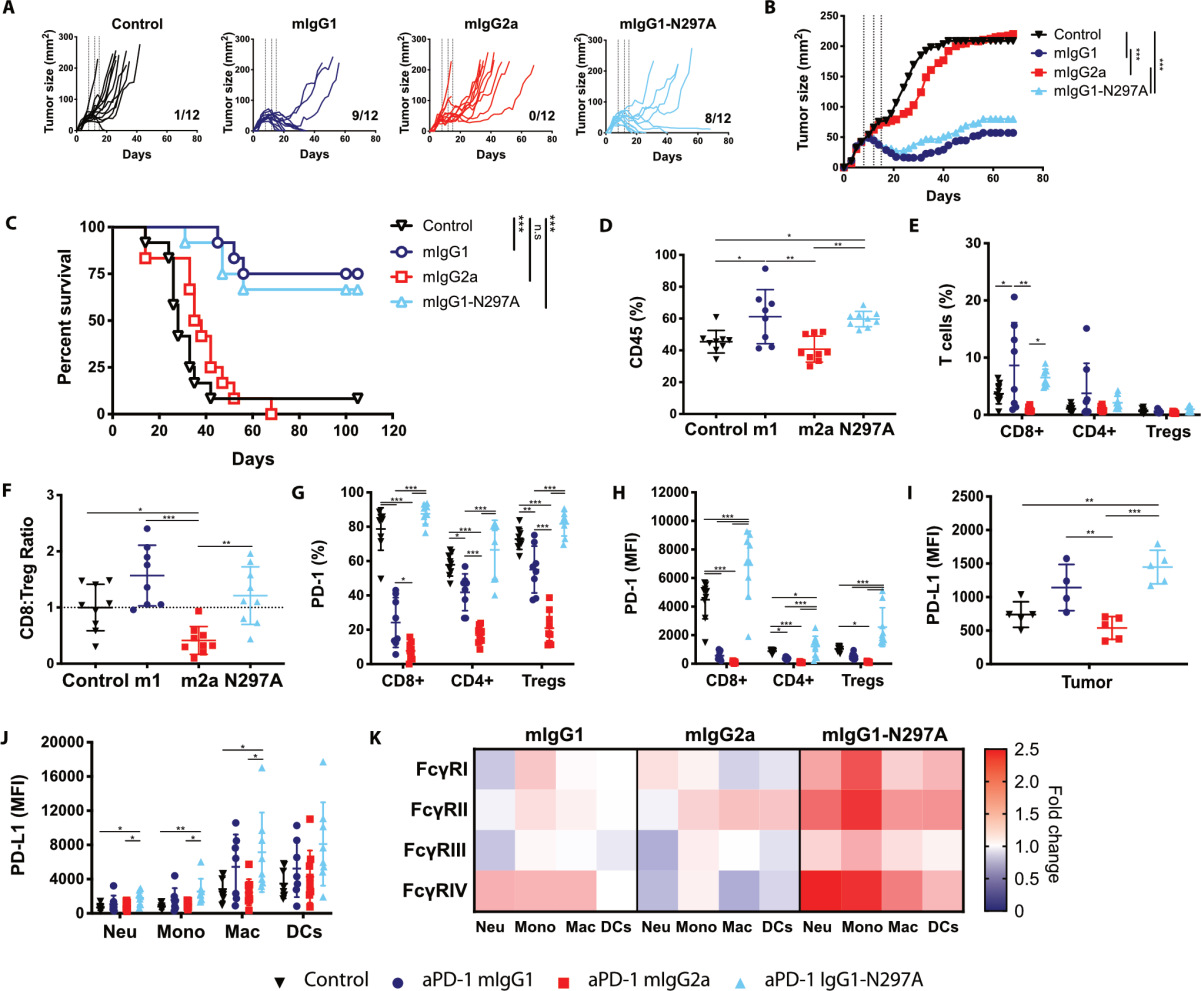
Anti-PD-1 mIgG1 and mIgG1-N297A augment antitumor immunity against MC38 tumors while mIgG2a abrogates therapeutic activity. C57BL/6 mice received 5×10^5^ MC38 cells s.c. on day 0. On days 8, 12, and 15 mice received 200 µg (i.p) anti-PD-1 isotypes or irrelevant mAbs. Tumor growth was monitored and mice culled when mean tumor area exceeded 225 mm^2^. Data are presented as tumor area (mm^2^) for each individual mouse (A) or the mean of the group (B). C) Kaplan-Meier curves showing percentage survival to humane end point on days after tumor inoculation. Experiment performed twice, N=12 mice per group. Log-rank (Mantel-Cox) Test, ***p<0.001. (D–K) Mice were sacrificed on day 16 and spleen and tumor analyzed by flow cytometry. (D, E) Frequency of CD45+ immune infiltrates (D) and T lymphocyte populations (expressed as % of CD45+ cells) (E). (F) CD8:Treg ratios expressed as fold change compared with control mice. (G, H) Expression of PD-1 on T lymphocyte populations as % (G) or MFI (H). (I, J) Expression of PD-L1 on tumor cells (I) or myeloid infiltrating subpopulations (J) presented as MFI. (K) Heat map indicating relative expression of FcγRs in treatment groups compared with controls. Colors represent the mean ratio of a group, where 1=no change; 1<downregulation; and 1>upregulation relative to controls. Experiment performed twice, N=7–9 mice per group. Bars represent mean±SD.D, *p<0.05, **p<0.01, ***p<0.001 (one-way ANOVA). ANOVA, analysis of variance; FcγRs, Fcγ receptors; MFI, mean fluorescence intensity; PD-1, programmed cell-death; s.c., subcutaneously.

Having established the role of anti-PD-1 isotype in an immunization setting, we sought to determine how this might impact antitumor immunity. In the immunologically ‘hot’ MC38 tumor model,^19 35^ monotherapy with mIgG1-N297A significantly decreased tumor growth and improved overall survival (figure 5A–C). Interestingly, and contrary to previous reports using the same model,^17^ mIgG1 had an equivalent therapeutic activity to mIgG1-N297A, while mIgG2a was completely ineffective (figure 5A–C). Monotherapy with both anti-PD-1 mIgG1 and mIgG1-N297A led to long-term antitumor immunity, with survivors able to reject tumor rechallenge (online supplemental figure S4).

To better understand what changes were driving these therapeutic differences, we sought to investigate the magnitude and phenotype of immune infiltrates in MC38 tumors (online supplemental figure S5 and S6). Results showed that both mIgG1 and mIgG1-N297A increased the overall percentage of CD45+ immune infiltrate (figure 5D) and the percentage of CD8 TILs (figure 5E). In contrast, mIgG2a had the opposite effect, decreasing CD8 T-cell numbers and thereby the CD8:Treg ratio (figure 5F, online supplemental figure S7A).

**Figure 6:**
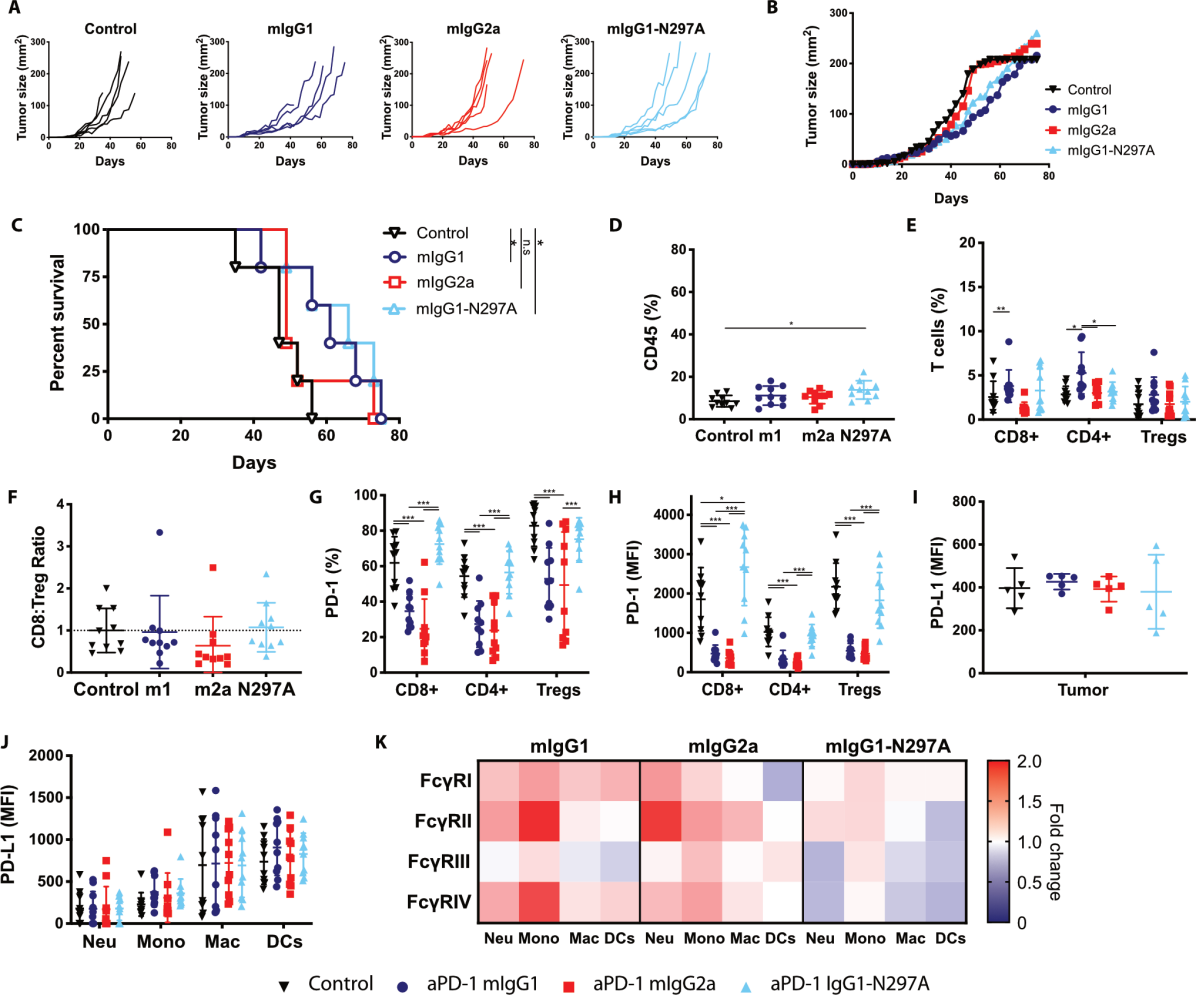
Similar Fc requirements for anti-PD-1 mAb therapy in cold tumors are not accompanied by improved long-term survival. C57BL/6 mice received 5×10^5^ 9464D cells s.c. on day 0. (A–C) When tumors became 5×5 mm mice received weekly doses of 200 µg (i.p) anti-PD-1 isotypes or irrelevant mAbs. Tumor growth was monitored and mice culled when mean tumor area exceeded 225 mm^2^. Data are presented as tumor area (mm^2^) for each individual mouse (A) or the mean of the group (B). (C) Kaplan-Meier curves showing percentage survival to humane end point on days after tumor inoculation. Experiment performed once, N=5 mice per group. Log-rank (Mantel-Cox) Test, *p<0.05. (D–K) When tumors became 7×7 mm, mice received three doses of 200 µg (i.p) anti-PD-1 mIgG1, mIgG2a, mIgG1-N297A or irrelevant mAbs on days 1, 5 and 8. Mice were sacrificed on day 9 and spleen and tumor analyzed by flow cytometry. (D, E) Frequency of CD45+ immune infiltrates (D) and T lymphocyte populations (expressed as % of CD45+ cells) (E). F) CD8:Treg ratios expressed as fold change compared with control mice. (G, H) Expression of PD-1 on T lymphocyte populations as % (G) or MFI (H). I–J) Expression of PD-L1 on tumor cells (I) or myeloid infiltrating subpopulations (J) presented as MFI. (K) Heat map indicating relative expression of FcγRs in treatment groups compared with controls. Colors represent the mean ratio of a group, where 1=no change; 1<downregulation; and 1>upregulation relative to controls. Experiment performed twice, n=10 mice per group. Bars represent mean±SD, *p<0.05, **p<0.01, ***p<0.001 (One-way ANOVA). ANOVA, analysis of variance; i.p, intraperitoneally; mAb, monoclonal antibodie; MFI, mean fluorescence intensity; ns, not significant; PD-1, programmed cell-death; s.c., subcutaneously.

Among CD8 TILs, approximately 80% expressed high levels of PD-1 in control mice, likely marking a population subject to chronic antigen stimulation (figure 5G, H). Moreover, the highest expression of PD-1 was found on CD8 TILs, implying that these T cells would be the primary target population of anti-PD-1 in this model. Alongside the significant upregulation of PD-1 on TILs (figure 5H), monotherapy with mIgG1-N297A increased PD-1 on T cells in the spleen (online supplemental figure S7C), demonstrating the ability of this isotype to induce systemic T-cell activation. In contrast, mIgG1 and mIgG2a strongly decreased PD-1 expression compared with controls (figure 5G, H). Mice treated with mIgG2a displayed the lowest percentage and absolute number of PD-1+ TILs (figure 5G, online supplemental figure S7B), supporting the contention that this isotype causes depletion of activated PD-1+ T cells. Although mIgG1 also decreased the number of PD-1+ TILs (figure 5G, online supplemental figure S7B), the parallel increase in CD8s could indicate that this isotype is less effective at depleting PD-1+ T cells compared with mIgG2a, and instead may only lead to loss of PD-1 from the cell surface.^36 37^ Of note, mIgG1 displayed a higher degree of variability in CD8 T-cell infiltration compared with mIgG1-N297A (figure 5E, online supplemental figure S7A), further reinforcing that the activity of an Fc-competent anti-PD-1 may be strongly susceptible to subtle differences in immune environment. Supporting this, the percentage of CD8 T cells correlated with the A:I expression ratio of FcγRs in tumor-infiltrating myeloid cells (online supplemental figure S7D).

During the course of an immune response, activated T cells release pro-inflammatory cytokines such as IFN-γ that can up-regulate PD-L1 on tumor and host cells.^38–40^ The increase in PD-L1 expression in MC38 cells after therapy with mIgG1 and mIgG1-N297A (figure 5I) might, therefore, reflect ongoing immune activation with secretion of inflammatory cues in the TME. Notably, mIgG1-N297A also led to an upregulation of PD-L1 on all myeloid subsets (figure 5J), suggestive of their activation.^41^ Beyond PD-L1, myeloid cells also express a complex repertoire of activating and inhibitory FcγRs, the relative expression of which can give an insight into their activation state.^42^ Treatment with anti-PD-1 mIgG1-N297A induced a clear upregulation of FcγRs on myeloid cells, whereas only a modest increase in the medium-to-high affinity activating FcγRIV was noted in the mIgG1 treatment group (figure 5K). Importantly, levels of FcγRIV on macrophages correlated with antitumor activity by anti-PD-1 mAbs (online supplemental figure S7E), evidencing the impact of myeloid phenotype on PD-1 blockade responsiveness. Overall, these results highlight the ability of anti-PD-1 mIgG1-N297A to indirectly modulate tumor-infiltrating myeloid cells and favor the acquisition of a more activated phenotype.

### Anti-PD-1 mAbs retain equivalent Fc requirements in immunologically cold tumors despite decreased efficacy

To investigate the Fc requirements of anti-PD-1 in a less responsive tumor model, we used the immunologically ‘cold’ 9464D pediatric neuroblastoma line.^20 21^ Despite minimal therapeutic activity, limited to a delay in tumor growth (figure 6A–B), treatment with mIgG1 and mIgG1-N297A resulted in improved survival compared with controls (figure 6C), thereby displaying the same isotype dependent effects as those observed in the responsive MC38. In keeping with previous findings, treatment with mIgG1-N297A resulted in increased immune infiltration in 9464D tumors (figure 6D), although this remained below 20%. In addition, mIgG1 and mIgG1-N297A tended to increase CD8 and CD4 TILs (figure 6E, online supplemental figure S8A), while mIgG2a showed a clear trend towards a decreased CD8:Treg ratio (figure 6F).

Notably, despite the decreased magnitude of therapeutic response, PD-1 expression exhibited the same isotype dependent changes to that in MC38, with increased PD-1 levels induced by mIgG1-N297A but decreased expression after mIgG1 and mIgG2a treatment (figure 6G–H) in the tumor and increased PD-1 on T-cell subsets in the spleen (online supplemental figure S8C). In neuroblastoma tumors, however, a significant depletion of PD-1+ CD8 TILs was not noted (online supplemental figure S8B) perhaps due to the already low absolute numbers of TILs compared with MC38. These results suggest that anti-PD-1 mAbs can have systemic effects and induce similar changes in immunologically cold tumors with low TMB, as exemplified by the 9464D model, despite limited impact on tumor growth and survival.

Despite these immune-modulatory effects, PD-1 blockade did not alter PD-L1 expression in 9464D cells or myeloid subsets, nor did mIgG1-N297A modulate myeloid FcγR expression (figure 6I–K). Although there was a trend towards a correlation between CD8 T-cell infiltration and A:I FcγR ratio (online supplemental figure S8D), FcγRIV expression did not correlate with antitumor activity in 9464D as observed in MC38. This reduced immune modulation by anti-PD-1 in 9464D tumors likely reflects the low numbers of TILs, which in turn impairs the secondary inflammatory effect on other populations within the TME and overall therapeutic activity.

## Discussion

Elucidating the mechanisms that affect response to PD-1 blocking mAbs is of critical importance, given the increasing number of trials evaluating these agents for cancer immunotherapy. Previous work highlighted the potentially detrimental role that FcγR engagement by anti-PD-1 mAbs could have in preclinical models,^17^ although strong evidence is still lacking. In addition, further work is required to translate preclinical results using mouse mAbs into the human system. Here, in multiple immune settings, we found that engineered Fc-null anti-PD-1 mAbs are the optimal format to induce effective T-cell immunity.

In an immunization setting with the model antigen OVA, anti-PD-1 mIgG1-N297A enhanced the expansion of adoptively transferred OT-I cells, outperforming mIgG1 and mIgG2a. Despite reduced efficacy, mIgG1 demonstrated some activity when compared with mIgG2a. This is possibly due to the high A:I ratio of mIgG2a and its increased ability to trigger effector mechanisms, leading to ADCP of PD-1+ T cells. Moreover, mIgG1-N297A favored the acquisition of an effector-like phenotype in non-SIINFEKL specific T cells, suggesting that effective PD-1 blockade could broaden the immune response to other specificities. Importantly, these results were fully recapitulated in an endogenous setting, without adoptive transfer of high affinity OT-I cells, demonstrating their physiological relevance. To validate the translational potential of our findings, we compared the efficacy of a clinically relevant anti-PD-1 hIgG4 against an engineered Fc-null version in mice bearing hFcγRs. Following immunization with OVA, the human Fc-null anti-PD-1 outcompeted its Fc-competent counterpart, resulting in enhanced SIINFEKL-specific CD8 expansion and acquisition of effector phenotype in tetramer negative cells, recapitulating the murine data.

In contrast to OVA-specific immunizations, tumors exhibit numerous immunosuppressive mechanisms that collectively dampen antitumor immunity. Owing to its known sensitivity to immunotherapy,^35^ MC38 provides an ideal model to investigate differences among anti-PD-1 isotypes. In contrast to prior work suggesting that FcγRII engagement by mIgG1 reduced antitumor efficacy,^17^ both mIgG1 and mIgG1-N297A boosted T-cell infiltration, inducing significant and comparable long-term antitumor responses in MC38-bearing mice. In contrast, administration of anti-PD-1 mIgG2a completely abrogated the therapeutic effect of PD-1 blockade. This was likely caused by the depletion of PD-1+ CD8 TILs, as eluded to in previous work,^17^ and is further supported by our in vitro data demonstrating that anti-PD-1 mIgG2a can lead to phagocytosis of activated T cells. Notably, clinically relevant anti-PD-1 human mAbs were able to trigger in vitro phagocytosis of PD-1-transfected cells by macrophages,^18^ illustrating the parallels between mouse and human results.

Previous reports identified a subset of PD-1+ TILs that proliferate in response to PD-1 blockade, giving rise to PD-1^Hi^ effector cells.^43^ Therefore, the upregulation of PD-1 noted on mIgG1-N297A treatment might mark the differentiation of such tumor-reactive subset of TILs. In contrast, mIgG1 treatment decreased PD-1 expression in all TILs. While this could reflect deletion of PD-1+ TILs via engagement of activating FcγRs, it is important to note that some mice still demonstrated increased infiltration of CD8 TILs. Alternatively, binding to FcγRII/III on macrophages by anti-PD-1 has been shown to cause removal of mAb from the T-cell surface.^44^ It is therefore plausible that, due to the high affinity interaction between PD-1 and anti-PD-1 mAbs, removal of the antibody is accompanied by removal of the receptor, while sparing T-cell integrity. In favor of this, a similar decrease in CD25 expression (but not Treg deletion) was observed following anti-CD25 rat IgG1 therapy,^34^ which bears a comparable FcγR binding profile to mIgG1 (online supplemental figure S1). Taken together, these data point to a shared phenomenon across different T-cell receptors on treatment with mAbs with low A:I ratios and calls for caution when interpreting receptor expression data.

Despite the accepted view that PD-1 blocking mAbs predominantly work in cancers with favorable immunological and biological traits, there is a paucity of studies directly comparing the immune response in responsive vs non-responsive tumors. To perform such comparison, we used the 9464D neuroblastoma cell line bearing low TMB^21^ and scarce T-cell infiltration^20^ compared with MC38. Although anti-PD-1 displayed similar Fc requirements, with mIgG1 and mIgG1-N297A showing a trend towards improved efficacy, the overall therapeutic activity was minimal. 9464D TILs demonstrated similar changes to those in MC38, but the modulation of the myeloid compartment by mIgG1-N297A was lost. This might indicate that the absolute number of TILs is too low to be able to activate myeloid cells following PD-1 blockade, and hence unable to promote a shift to a more pro-inflammatory TME. As an alternative mechanism of primary resistance, we noted a similar level of PD-1 expression on CD8 TILs and Tregs in neuroblastoma tumors in contrast to MC38, where CD8 TILs had the highest expression. Interestingly, a recent report described that the balance of PD-1 expression between CD8 effectors and Tregs could predict the clinical efficacy of anti-PD-1.^45^ Our results agree with this hypothesis, highlighting the potential detrimental effect of enhancing Treg function by blocking PD-1. Further to this, mIgG1-N297A increased PD-1 expression in splenic T cells, with Tregs displaying the highest levels in both MC38 and neuroblastoma models. This poses the question as to whether systemic PD-1 blockade could also lead to the parallel activation of peripheral Tregs, providing an acquired mechanism of resistance. In support of this, a study found that the frequency of Ki67+ Tregs increased in TILs of hyperprogressive disease patients after anti-PD-1 therapy, and in vitro PD-1 blockade augmented Treg-mediated immunosuppressive activity.^46^

Here, we elucidate Fc:FcγR interactions as a key mechanism of primary resistance to PD-1 blockade therapy. The immune context-dependent nature of these interactions hinders the use of Fc-competent anti-PD-1 mAbs, which has implications for clinically approved huIgG4 antibodies, and highlights the potential benefits of using engineered Fc-null variants to widen patient responsiveness. Although the engineering of Fc-null anti-PD-1 alone was insufficient to sensitize non-responsive tumors to monotherapy, we envisage that Fc:FcγR interactions might provide a means of acquired resistance in non-responsive tumor types in the context of successful combination strategies that increase T-cell infiltration and PD-1 upregulation.

## Data Availability

All data relevant to the study are included in the article or uploaded as online supplemental information.

## References

[1] Topalian SL, Drake CG, Pardoll DM. Targeting the PD-1/B7-H1(PD-L1) pathway to activate anti-tumor immunity. Curr Opin Immunol 2012;24:207–12. 10.1016/j.coi.2011.12.009 22236695 PMC3319479

[2] Topalian SL, Hodi FS, Brahmer JR, et al. Safety, activity, and immune correlates of anti-PD-1 antibody in cancer. N Engl J Med 2012;366:2443–54. 10.1056/NEJMoa1200690 22658127 PMC3544539

[3] Hamid O, Robert C, Daud A, et al. Safety and tumor responses with lambrolizumab (anti-PD-1) in melanoma. N Engl J Med 2013;369:134–44. 10.1056/NEJMoa1305133 23724846 PMC4126516

[4] Dahal LN, Roghanian A, Beers SA, et al. FcgammaR requirements leading to successful immunotherapy. Immunol Rev 2015;268:104–22. 10.1111/imr.12342 26497516

[5] Nimmerjahn F, Ravetch JV. Fcγ receptors as regulators of immune responses. Nat Rev Immunol 2008;8:34–47. 10.1038/nri2206 18064051

[6] Nimmerjahn F, Ravetch JV. Divergent immunoglobulin G subclass activity through selective Fc receptor binding. Science 2005;310:1510–2. 10.1126/science.1118948 16322460

[7] Uchida J, Hamaguchi Y, Oliver JA, et al. The innate mononuclear phagocyte network depletes B lymphocytes through Fc receptor-dependent mechanisms during anti-CD20 antibody immunotherapy. J Exp Med 2004;199:1659–69. 10.1084/jem.20040119 15210744 PMC2212805

[8] Minard-Colin V, Xiu Y, Poe JC, et al. Lymphoma depletion during CD20 immunotherapy in mice is mediated by macrophage FcgammaRI, FcgammaRIII, and FcgammaRIV. Blood 2008;112:1205–13. 10.1182/blood-2008-01-135160 18495955 PMC2515149

[9] Montalvao F, Garcia Z, Celli S, et al. The mechanism of anti-CD20-mediated B cell depletion revealed by intravital imaging. J Clin Invest 2013;123:5098–103. 10.1172/JCI70972 24177426 PMC3859399

[10] Li F, Ravetch JV. Inhibitory Fcgamma receptor engagement drives adjuvant and anti-tumor activities of agonistic CD40 antibodies. Science 2011;333:1030–4. 10.1126/science.1206954 21852502 PMC3164589

[11] Li F, Ravetch JV. Apoptotic and antitumor activity of death receptor antibodies require inhibitory Fcgamma receptor engagement. Proc Natl Acad Sci U S A 2012;109:10966–71. 10.1073/pnas.1208698109 22723355 PMC3390832

[12] White AL, Chan HTC, Roghanian A, et al. Interaction with FcgammaRIIB is critical for the agonistic activity of anti-CD40 monoclonal antibody. J Immunol 2011;187:1754–63. 10.4049/jimmunol.1101135 21742972

[13] Xu Y, Szalai AJ, Zhou T, et al. Fc gamma Rs modulate cytotoxicity of anti-Fas antibodies: implications for agonistic antibody-based therapeutics. J Immunol 2003;171:562–8. 10.4049/jimmunol.171.2.562 12847219

[14] Bruhns P, Iannascoli B, England P, et al. Specificity and affinity of human Fcgamma receptors and their polymorphic variants for human IgG subclasses. Blood 2009;113:3716–25. 10.1182/blood-2008-09-179754 19018092

[15] Hussain K, Hargreaves CE, Roghanian A, et al. Upregulation of FcgammaRIIb on monocytes is necessary to promote the superagonist activity of TGN1412. Blood 2015;125:102–10. 10.1182/blood-2014-08-593061 25395427

[16] Isaacs JD, Wing MG, Greenwood JD, et al. A therapeutic human IgG4 monoclonal antibody that depletes target cells in humans. Clin Exp Immunol 1996;106:427–33. 10.1046/j.1365-2249.1996.d01-876.x 8973608 PMC2200623

[17] Dahan R, Sega E, Engelhardt J, et al. FcγRs modulate the anti-tumor activity of antibodies targeting the PD-1/PD-L1 axis. Cancer Cell 2015;28:285–95. 10.1016/j.ccell.2015.08.004 26373277

[18] Zhang T, Song X, Xu L, et al. The binding of an anti-PD-1 antibody to FcgammaRIota has a profound impact on its biological functions. Cancer Immunol Immunother 2018;67:1079–90. 10.1007/s00262-018-2160-x 29687231 PMC6006217

[19] Efremova M, Rieder D, Klepsch V, et al. Targeting immune checkpoints potentiates immunoediting and changes the dynamics of tumor evolution. Nat Commun 2018;9:32. 10.1038/s41467-017-02424-0 29296022 PMC5750210

[20] Webb ER, Lanati S, Wareham C, et al. Immune characterization of pre-clinical murine models of neuroblastoma. Sci Rep 2020;10:16695. 10.1038/s41598-020-73695-9 33028899 PMC7541480

[21] Voeller J, Erbe AK, Slowinski J, et al. Combined innate and adaptive immunotherapy overcomes resistance of immunologically cold syngeneic murine neuroblastoma to checkpoint inhibition. J Immunother Cancer 2019;7:344. 10.1186/s40425-019-0823-6 31810498 PMC6898936

[22] Moreno-Vicente J, Beers SA, Gray JC. PD-1/PD-L1 blockade in paediatric cancers: what does the future hold? Cancer Lett 2019;457:74–85. 10.1016/j.canlet.2019.04.025 31055109

[23] Majzner RG, Simon JS, Grosso JF, et al. Assessment of programmed death-ligand 1 expression and tumor-associated immune cells in pediatric cancer tissues. Cancer 2017;123:3807–15. 10.1002/cncr.30724 28608950

[24] Chowdhury F, Dunn S, Mitchell S, et al. PD-L1 and CD8 ^+^ PD1 ^+^ lymphocytes exist as targets in the pediatric tumor microenvironment for immunomodulatory therapy. Oncoimmunology 2015;4:e1029701. 10.1080/2162402X.2015.1029701

[25] Gillis CM, Jönsson F, Mancardi DA, et al. Mechanisms of anaphylaxis in human low-affinity IgG receptor locus knock-in mice. J Allergy Clin Immunol 2017;139:e14:1253–65. 10.1016/j.jaci.2016.06.058 27568081

[26] Xu D, Alegre ML, Varga SS, et al. In vitro characterization of five humanized OKT3 effector function variant antibodies. Cell Immunol 2000;200:16–26. 10.1006/cimm.2000.1617 10716879

[27] Hogquist KA, Jameson SC, Heath WR, et al. T cell receptor antagonist peptides induce positive selection. Cell 1994;76:17–27. 10.1016/0092-8674(94)90169-4 8287475

[28] Buchan SL, Dou L, Remer M, et al. Antibodies to costimulatory receptor 4-1BB enhance anti-tumor immunity via T regulatory cell depletion and promotion of CD8 T cell effector function. Immunity 2018;49:958–70. 10.1016/j.immuni.2018.09.014 30446386

[29] White AL, Chan HTC, French RR, et al. Conformation of the human immunoglobulin G2 hinge imparts superagonistic properties to immunostimulatory anticancer antibodies. Cancer Cell 2015;27:138–48. 10.1016/j.ccell.2014.11.001 25500122 PMC4297290

[30] Beers SA, French RR, Chan HTC, et al. Antigenic modulation limits the efficacy of anti-CD20 antibodies: implications for antibody selection. Blood 2010;115:5191–201. 10.1182/blood-2010-01-263533 20223920

[31] Tipton TRW, Roghanian A, Oldham RJ, et al. Antigenic modulation limits the effector cell mechanisms employed by type I anti-CD20 monoclonal antibodies. Blood 2015;125:1901–9. 10.1182/blood-2014-07-588376 25631769

[32] Tutt AL, James S, Laversin SA, et al. Development and characterization of monoclonal antibodies specific for mouse and human Fcγ receptors. J Immunol 2015;195:5503–16. 10.4049/jimmunol.1402988 26512139

[33] Arduin E, Arora S, Bamert PR, et al. Highly reduced binding to high and low affinity mouse Fc gamma receptors by L234A/L235A and N297A Fc mutations engineered into mouse IgG2a. Mol Immunol 2015;63:456–63. 10.1016/j.molimm.2014.09.017 25451975

[34] Arce Vargas F, Furness AJS, Solomon I, et al. Fc-optimized anti-CD25 depletes tumor-infiltrating regulatory T cells and synergizes with PD-1 blockade to eradicate established tumors. Immunity 2017;46:577–86. 10.1016/j.immuni.2017.03.013 28410988 PMC5437702

[35] Garris CS, Arlauckas SP, Kohler RH, et al. Successful anti-PD-1 cancer immunotherapy requires T Cell-Dendritic cell crosstalk involving the cytokines IFN-γ and IL-12. Immunity 2018;49:1148–61. 10.1016/j.immuni.2018.09.024 30552023 PMC6301092

[36] Beum PV, Kennedy AD, Williams ME, et al. The shaving reaction: rituximab/CD20 complexes are removed from mantle cell lymphoma and chronic lymphocytic leukemia cells by THP-1 monocytes. J Immunol 2006;176:2600–9. 10.4049/jimmunol.176.4.2600 16456022

[37] Dahal LN, Huang C-Y, Stopforth RJ, et al. Shaving is an epiphenomenon of type I and II anti-CD20-mediated phagocytosis, whereas antigenic modulation limits type I monoclonal antibody efficacy. J Immunol 2018;201:1211–21. 10.4049/jimmunol.1701122 29997125 PMC6082343

[38] Taube JM, Anders RA, Young GD, et al. Colocalization of inflammatory response with B7-H1 expression in human melanocytic lesions supports an adaptive resistance mechanism of immune escape. Sci Transl Med 2012;4:127ra37. 10.1126/scitranslmed.3003689 PMC3568523 22461641

[39] Abiko K, Matsumura N, Hamanishi J, et al. IFN-γ from lymphocytes induces PD-L1 expression and promotes progression of ovarian cancer. Br J Cancer 2015;112:1501–9. 10.1038/bjc.2015.101 25867264 PMC4453666

[40] Xiao W, Klement JD, Lu C, et al. IFNAR1 controls autocrine type I IFN regulation of PD-L1 expression in myeloid-derived suppressor cells. J Immunol 2018;201:264–77. 10.4049/jimmunol.1800129 29752314 PMC6008224

[41] Kondo A, Yamashita T, Tamura H, et al. Interferon-gamma and tumor necrosis factor-alpha induce an immunoinhibitory molecule, B7-H1, via nuclear factor-kappaB activation in blasts in myelodysplastic syndromes. Blood 2010;116:1124–31. 10.1182/blood-2009-12-255125 20472834 PMC3375140

[42] Dahal LN, Dou L, Hussain K, et al. STING activation reverses lymphoma-mediated resistance to antibody immunotherapy. Cancer Res 2017;77:3619–31. 10.1158/0008-5472.CAN-16-2784 28512240 PMC5500176

[43] Siddiqui I, Schaeuble K, Chennupati V, et al. Intratumoral Tcf1^+^PD-1^+^CD8^+^ T cells with stem-like properties promote tumor control in response to vaccination and checkpoint blockade immunotherapy. Immunity 2019;50:e10:195–211. 10.1016/j.immuni.2018.12.021 30635237

[44] Arlauckas SP, Garris CS, Kohler RH, et al. In vivo imaging reveals a tumor-associated macrophage-mediated resistance pathway in anti-PD-1 therapy. Sci Transl Med 2017;9 10.1126/scitranslmed.aal3604 28490665 PMC5734617

[45] Kumagai S, Togashi Y, Kamada T, et al. The PD-1 expression balance between effector and regulatory T cells predicts the clinical efficacy of PD-1 blockade therapies. Nat Immunol 2020;21:1346–58. 10.1038/s41590-020-0769-3 32868929

[46] Kamada T, Togashi Y, Tay C, et al. PD-1^+^ regulatory T cells amplified by PD-1 blockade promote hyperprogression of cancer. Proc Natl Acad Sci U S A 2019;116:9999–10008. 10.1073/pnas.1822001116 31028147 PMC6525547

