# Supplementary material for: Fc-null anti-PD-1 monoclonal antibodies deliver optimal checkpoint blockade in diverse immune environments
Source: J Immunother Cancer. 2022 Jan 11;10(1):e003735. doi: 10.1136/jitc-2021-003735 (PMC8753441; doi:10.1136/jitc-2021-003735)
Supplement: Supplementary data [file jitc-2021-003735supp001.pdf]

Supplementary Materials

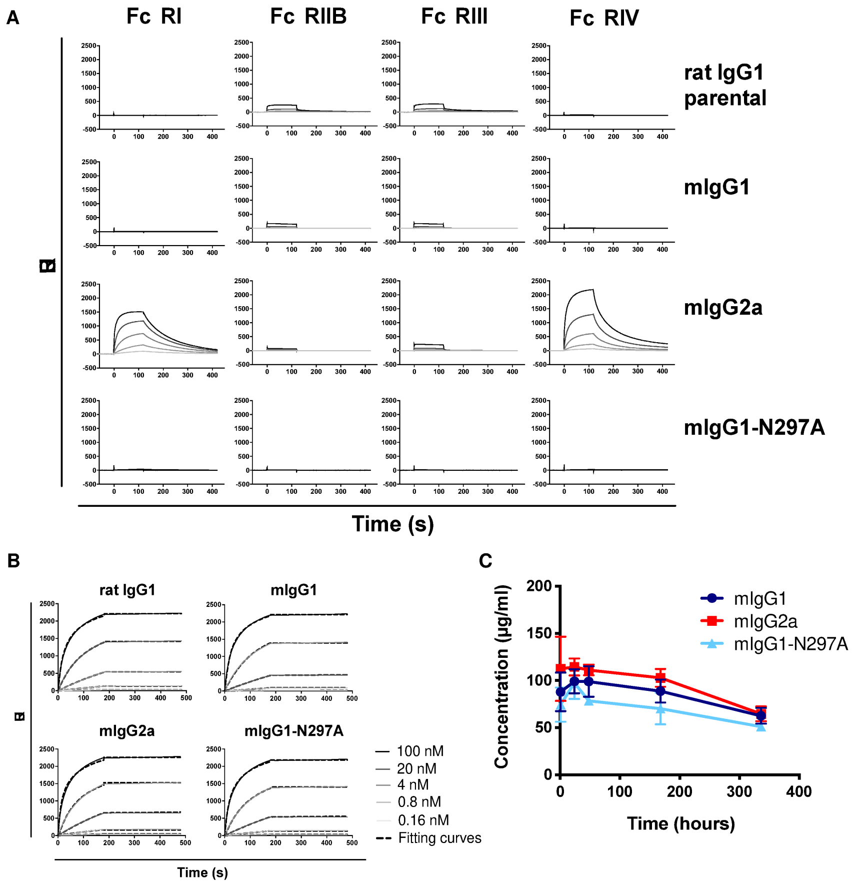

aPD-1 mAbs	K_{on}	K_{off}	K_D (K_{off}/ K_{on})
rIgG1	1.8×10^{5}	1.06×10^{-7}	5.88×10^{-13}
mIgG1	2.4×10^{5}	8.86×10^{-7}	3.69×10^{-12}
mIgG2a	2.4×10^{5}	7.3×10^{-8}	3.22×10^{-13}
mIgG1-N297A	1.9×10^{5}	4.5×10^{-7}	2.36×10^{-12}

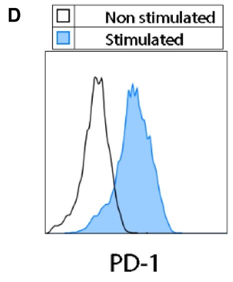

Fig. S1 Engineered mouse anti-PD-1 mAbs show expected in vitro binding and similar in vivo half-life.

A) Surface plasmon resonance analysis of anti-PD-1 (clone EW1-9) mIgG1, mIgG2a, mIgG1-N297A and the parental rIgG1 binding to mouse FcγRI, II III and IV. Soluble anti-

PD-1 mAbs (0, 6.2, 15.5, 55.5, 166.7 and 500 nM) were passed over recombinant His-tagged FcγRs immobilized at 1000 RU. B) Binding kinetics and avidity analysis of anti-PD-1 mAbs to mouse PD-1. Soluble anti-PD-1 mAbs (0.16, 0.8, 4, 20 and 100 nM) were passed over recombinant His-tagged PD-1 (2000 RU). A bivalent fitting model was applied to calculate association (Kon) and dissociation (Koff) constants. KD values calculated from Kon and Koff represent the predicted avidity of mAbs towards murine PD-1 protein. C) C57BL/6 WT mice were intraperitoneally injected with 250 µg of anti-PD-1 murine mAbs and serum samples were collected at 1h, 24h, 48h, 7 and 14 days. Detection of anti-PD-1 mAb-binding to PD-1-expressing HEK293F cells was performed by PE-labelled secondary antibody staining. MAb concentrations in serum were obtained by interpolation of a standard curve. Experiments were performed once. Bars represent mean ± S.D, 3 mice per treatment group. D) Histogram illustrating up-regulation of PD-1 on splenic T cells activated with 5 µg/ml plate-bound OKT3 for 48h.

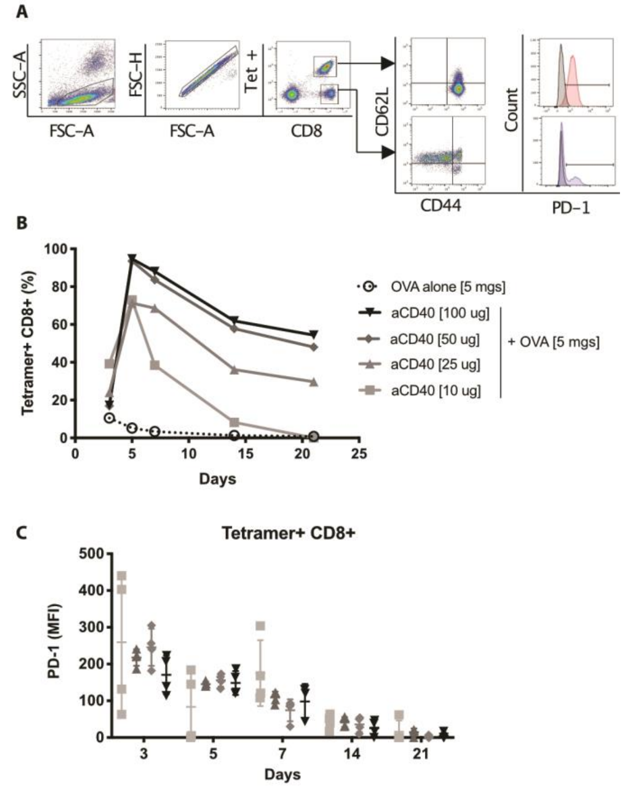

Fig. S2 Anti-CD40 mAbs stimulate OT-I expansion and PD-1 up-regulation.

A) Gating strategy followed to quantify SIINFEKL-specific CD8 T-cell responses in peripheral blood and T-cell phenotype in terms of CD62L, CD44 and PD-1 expression by flow cytometry. B-C) Groups of C57BL/6 WT mice received OT-I cell transfer prior to intraperitoneal injection with 5 mg OVA and the indicated amounts of anti-CD40 mAb (clone 3/23 mIgG1) i.p. on day 0. The percentage (B) and PD-1 expression (C) of SIINFEKL-specific CD8 T cells at the indicated timepoints after treatment are shown. Due to the low percentage of SIINFEKL-specific CD8 T cells in mice treated with OVA alone, PD-1 expression is not shown for this group. Bars represent mean ± S.D. Experiment performed once, N=4 mice per group.

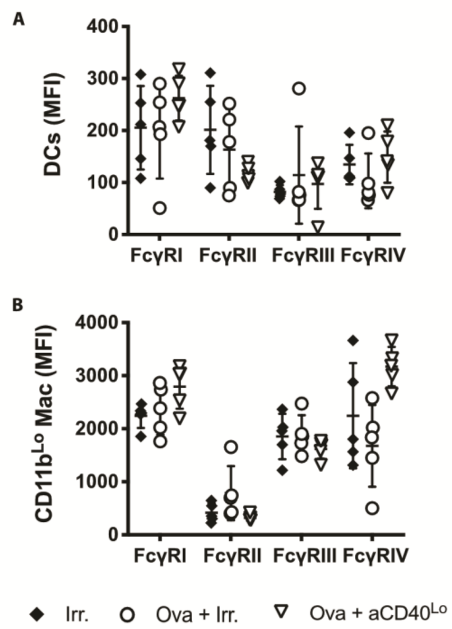

Fig. S3 Treatment with ovalbumin and anti-CD40 mAb modulates FcγRs in myeloid cells.

A-B) Groups of C57BL/6 WT mice received 10 µg irrelevant mAb alone; 10 µg irrelevant mAb plus 5 mg OVA; or 100 µg anti-CD40 mAb plus 5 mg OVA. Spleens were harvested 3 days after treatment. Expression of mouse FcγRI, II III and IV shown as mean fluorescence intensity in DCs (CD11b+, F4/80Lo, Ly6G-, Ly6C-, CD11c+, MHC-II+) (A) and tissue-resident macrophages (CD11bLo, F4/80Hi) (B). Experiment performed once, N=5 mice per group. Bars represent mean ± S.D (One-way ANOVA).

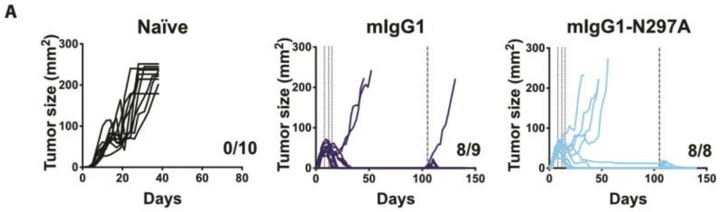

Fig. S4 Anti-PD-1 mIgG1 and Fc-null mAbs protect against MC38 re-challenge.

Groups of C57BL/6 WT mice received 5 x 105 MC38 cells s.c. on day 0. On days 8, 12, and 15 mice received 200 µg (i.p) anti-PD-1 mIgG1, mIgG2a, mIgG1-N297A or irrelevant mAbs. Tumor growth was monitored and mice culled when mean tumor area exceeded 225 mm2. Long term (100 days) surviving mice and naïve controls were re-challenged with 5 x 105 MC38 cells s.c. and tumor growth curves to humane end-point plotted for individual mice (n=8-10 mice per group).

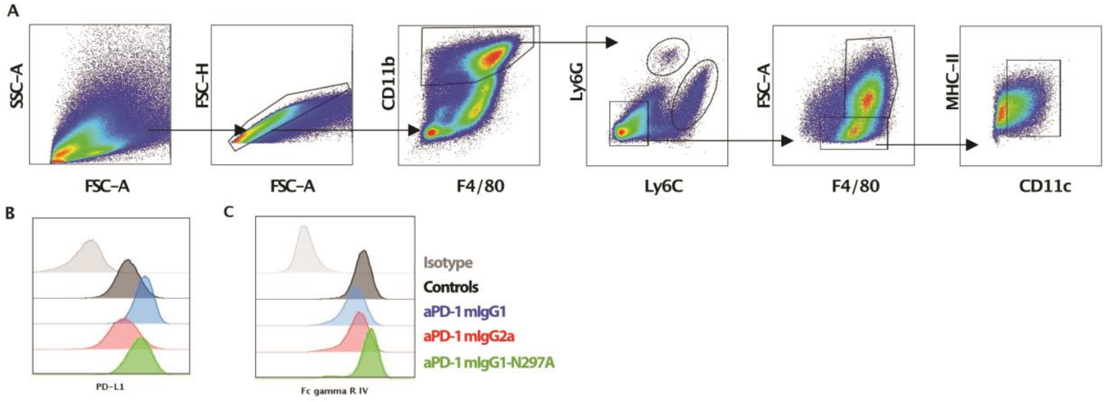

Fig. S5 Gating strategy followed to depict lymphocyte populations in tumors and spleens.

Groups of C57BL/6 WT mice received 5 x 105 MC38 cells s.c. on day 0. On days 8, 12, and 15 mice received 200 µg (i.p) anti-PD-1 mIgG1, mIgG2a, mIgG1-N297A or irrelevant mAbs. Mice were sacrificed on day 16 and spleen and tumor analyzed by flow cytometry. A) Gating strategy followed to analyze lymphocyte populations in spleen and tumor. T-cell populations gated included CD8+, CD4+FoxP3- (effector CD4 T cells) and CD4+FoxP3+ (Tregs). B) Representative histogram plots showing the expression of PD-1 (B) on tumor-infiltrating CD8+ lymphocytes.

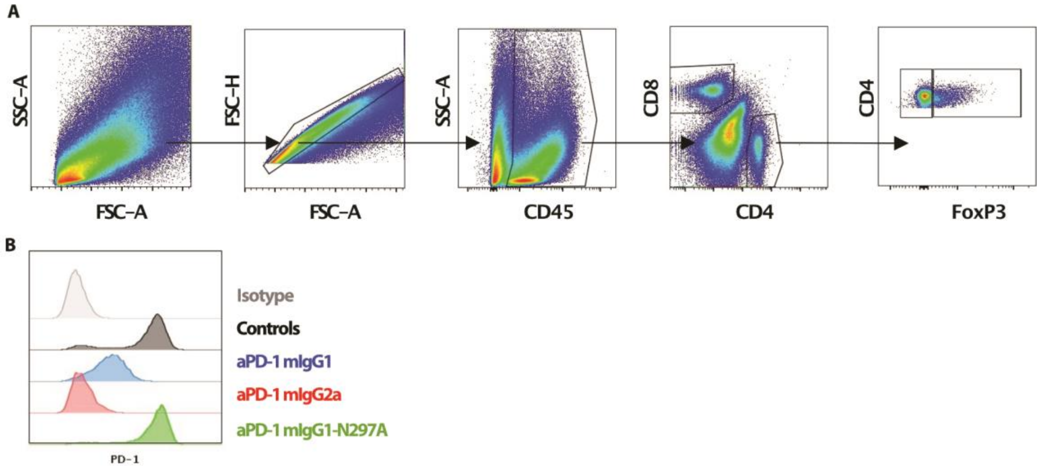

Fig. S6 Gating strategy followed to depict myeloid populations in tumors and spleens.

Groups of C57BL/6 WT mice received 5 x 105 MC38 cells s.c. on day 0. On days 8, 12, and 15 mice received 200 µg (i.p) anti-PD-1 mIgG1, mIgG2a, mIgG1-N297A or irrelevant mAbs. Mice were sacrificed on day 16 and spleen and tumor analyzed by flow cytometry. A) Gating strategy followed to analyze myeloid infiltrates in spleen and tumor. Myeloid populations gated included neutrophils (CD11b+, Ly6gHi, Ly6C+), monocytes (CD11b+, Ly6gLo, Ly6C+), macrophages (CD11b+, F4/80Hi) and DCs (CD11b+, F4/80Lo, MHC-II+, CD11c+). B-C) Representative histogram plots showing the expression of PD-L1 (B) and (C) FcγRIV on tumor-infiltrating macrophages.

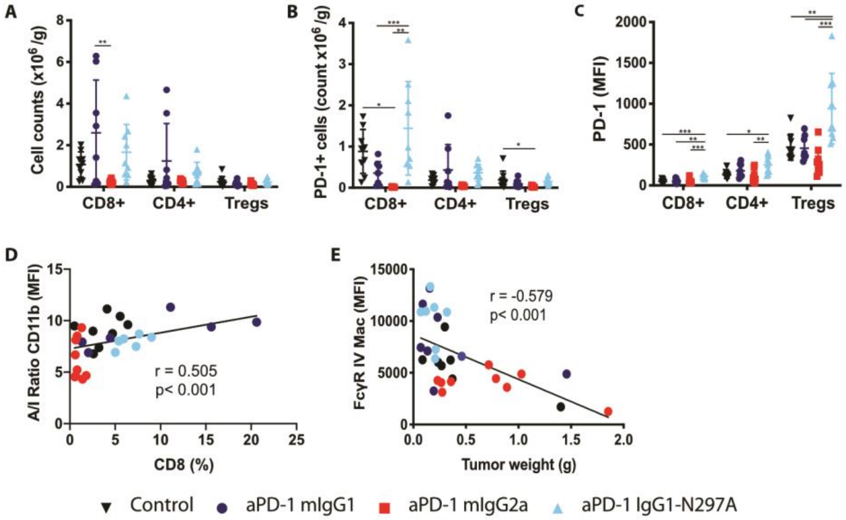

Fig. S7 Anti-PD-1 mIgG1 and Fc-null mAbs induce local and systemic immune activation in MC38-bearing mice.

Groups of C57BL/6 WT mice received 5 x 105 MC38 cells s.c. on day 0. On days 8, 12, and 15 mice received 200 µg (i.p) anti-PD-1 mIgG1, mIgG2a, mIgG1-N297A or irrelevant mAb controls. Mice were sacrificed on day 16 and spleen and tumor analyzed by flow cytometry. A-B) Cell counts of lymphocyte populations (A) and PD-1+ subpopulations (B) at the tumor. C) Expression of PD-1 expressed as mean fluorescence intensity on lymphocytes at the spleen. D-E) Correlation between A/I ratio in myeloid cells and percentage of CD8 (D) or between FcγRIV expression in macrophages and tumor weight (E). Pearson coefficient and p value (two-tailed) were calculated for each pair. Experiment performed twice, N=8-9 mice per group. Bars represent mean ± S.D, ***$p<0.001$, **$p<0.01$, *$p<0.05$ (One-way ANOVA).

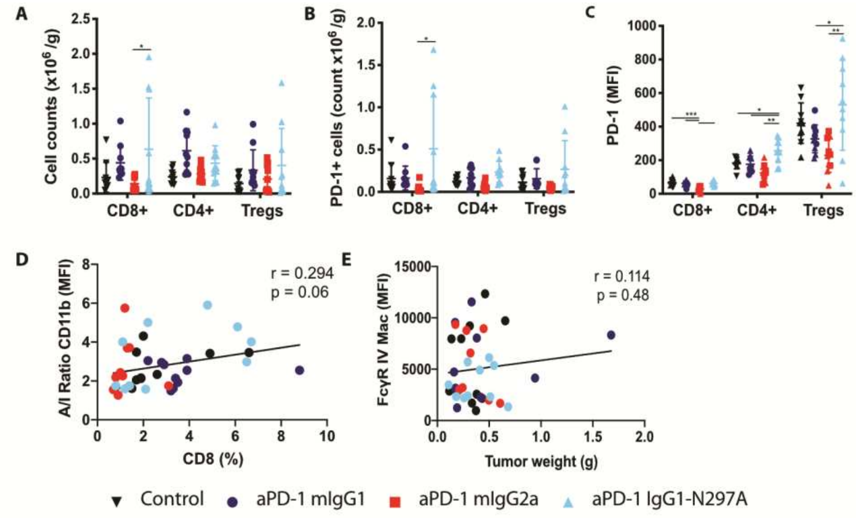

Fig. S8 Anti-PD-1 mIgG1 and Fc-null mAbs show a trend towards immune activation but no correlation with anti-tumor activity in 9464D.

Groups of C57BL/6 WT mice received 5 x 105 9464D cells s.c. on day 0. When tumors became 7x7 mm, mice received three doses of 200 µg (i.p) anti-PD-1 mIgG1, mIgG2a, mIgG1-N297A or irrelevant mAb controls on days 1, 5 and 8. Mice were sacrificed on day 9 and spleen and tumor analyzed by flow cytometry. A-B) Cell counts of lymphocyte populations (A) and PD-1+ subpopulations (B) at the tumor. C) Expression of PD-1 expressed as mean fluorescence intensity on lymphocytes at the spleen. D-E) Correlation between A/I ratio in myeloid cells and percentage of CD8 (D) or between FcγRIV expression in macrophages and tumor weight (E). Pearson coefficient and p value (two-tailed) were calculated for each pair. Experiment performed twice, N=10 mice per group. Bars represent mean ± S.D, ***p<0.001, **p<0.01, *p<0.05 (One-way ANOVA).
